# Hypoxia and HIF Signaling: One Axis with Divergent Effects

**DOI:** 10.3390/ijms21165611

**Published:** 2020-08-05

**Authors:** Chiara Corrado, Simona Fontana

**Affiliations:** Department of Biomedicine, Neurosciences and advanced Diagnostics (Bi.N.D), University of Palermo, via Divisi 83, 90133 Palermo, Italy; simona.fontana@unipa.it

**Keywords:** hypoxia, HIF-α, immune cells, inflammation, acute and chronic diseases

## Abstract

The correct concentration of oxygen in all tissues is a hallmark of cellular wellness, and the negative regulation of oxygen homeostasis is able to affect the cells and tissues of the whole organism. The cellular response to hypoxia is characterized by the activation of multiple genes involved in many biological processes. Among them, hypoxia-inducible factor (HIF) represents the master regulator of the hypoxia response. The active heterodimeric complex HIF α/β, binding to hypoxia-responsive elements (HREs), determines the induction of at least 100 target genes to restore tissue homeostasis. A growing body of evidence demonstrates that hypoxia signaling can act by generating contrasting responses in cells and tissues. Here, this dual and controversial role of hypoxia and the HIF signaling pathway is discussed, with particular reference to the effects induced on the complex activities of the immune system and on mechanisms determining cell and tissue responses after an injury in both acute and chronic human diseases related to the heart, lung, liver, and kidney.

## 1. Introduction

The regulation and preservation of oxygen homeostasis has an important role in determining cell fate. Hypoxia refers to the availability of low or insufficient oxygen levels in a tissue and is associated with both physiological and pathological conditions.

Cells can adapt to oxygen deprivation induced by insufficient blood flow to specific organs, low levels of hemoglobin, or exposure to chemical compounds by modulating protein activity or acting at both the transcriptional and the post-transcriptional levels. The cellular response to hypoxia consists of the activation of multiple genes involved in different biological processes such as angiogenesis, glucose metabolism, and cell survival/proliferation [1].

The master regulator factor mediating the cellular response to this condition is the hypoxia-inducible factor (HIF). HIF is a family of transcription factors composed of a heterodimer of a constitutively expressed subunit, HIF-β, and an oxygen-regulated subunit, HIF-α [2]. The stability and activity of the α subunit of HIF are regulated by its post-translational modifications such as hydroxylation, which is discussed below. In particular, HIF regulation in response to hypoxia depends on the activity of prolyl-4-hydroxilases (PHDs) and factors inhibiting HIF-α (FIHs), which are responsible for stability and the full transcriptional activity of HIFs, respectively [2]. In normoxia, HIF-α subunits (HIF1-α, HIF2-α, or HIF3-α) remain inactive through hydroxylation in specific residues. In the presence of oxygen, the PHDs hydroxylate a specific proline residue of the HIF-α subunit (Pro564 on HIF1-α, Pro530 on HIF2-α, and Pro490 on HIF3-α), triggering a ubiquitination reaction by E3 ubiquitin ligase Von Hippel–Lindau protein (pVHL) and proteasome-mediated degradation [3,4,5].

HIF-α subunits can be hydroxylated by the FIHs proteins in proline and asparagine residues (Asn803 on HIF1-α and Asn851 on HIF2-α). Through silencing of the HIF-α transactivation domain, this prevents the interaction between HIF-α and its coactivator protein p300/ cAMP response element-binding protein (CREB) [6,7].

Under hypoxic conditions, since the oxygen level is low, HIF-α hydroxylation is inhibited. Consequently, the HIF-α subunit is translocated to the nucleus where it dimerizes with the HIF-β subunit; the heterodimeric complex binds to the specific DNA binding regions (hypoxia-responsive elements, or HREs) of its target genes, resulting in their transcriptional regulation [8]. This interaction determines the induction of at least 100 target genes such as erythropoietin (EPO) and vascular endothelial growth factor (VEGF) [9,10]. This results in the activation of several hypoxia adaptive pathways to restore tissue homeostasis, such as those mediated by nuclear factor-κB (NFκB) and Toll-like receptors (TLRs) [11,12].

Recent evidence indicates that the PI3K/Protein kinase B (Akt) pathway and the protein kinase A (PKA) pathway participate in HIF regulation. HIF1-α phosphorylation, mediated by PKA, directly affects its stabilization, inhibiting its proteasomal degradation [13,14,15]. Furthermore, the MAPK/ERK pathway promotes HIF1-α nuclear accumulation [16]. Several studies focused on the effects of Reactive Oxygen Species (ROS) on HIF-α regulation, highlighting a controversial role depending on the cellular and experimental model. Some evidence indicates that ROS can inhibit PHDs activity, thus increasing HIF1-α stability; however, other evidence supported the role of ROS in inducing the HIF1-α degradation by the ubiquitin proteasome pathway [17,18,19].

The relationship between hypoxia and cancer is a major focus of scientific research, and the modulating effects of HIF signaling activation on cancer cells have been widely analyzed by both original papers and reviews [20,21,22]. Here, we focus on the dual and controversial role that hypoxia and the HIF signaling pathway can have on different systems, specifically in terms of modulating the immune response and the progression of acute or chronic human diseases related to specific organs such as the heart, lung, liver, and kidney. The complexity of effects mediated by hypoxia and HIFs will be discussed in the light of recent studies.

## 2. Dual Role of Hypoxia in Inflammation and Immune System Regulation

There is a direct link between hypoxia and inflammation. Hypoxia and inflammation have been described as “two sides of the same coin” [23]. It is widely accepted that hypoxia can induce inflammation. On the other side, it is also true that inflammatory conditions can generate HIF signaling activation, as observed during bacterial infection [24,25].

Lopez-Pascual et al. demonstrated that a conditioned medium of murine macrophages pretreated with lipopolysaccharide (LPS) caused the upregulation of pro-inflammatory genes, such as Interleukin 10 or Interleukin 1beta, and the upregulation of the HIF1-α subunit and NFκB p65 [26].

With regard to the main role of ROS in HIF1-α regulation and inflammatory responses, evidence indicates that mitochondrial ROS exerts a negative regulation of PHD activity. High levels of ROS are able to affect the catalytic domain of PDH2, thus impairing its activity, or it can induce the specific post-translational modifications that in turn inhibit PHD2, which promotes HIF activation [27,28].

Hypoxia signaling and HIF activation can have anti- and pro-inflammatory effects to regulate the activity of immune system cells. Immune cells, when subjected to inflammatory stimulation, change their metabolic activity to acquire a phenotype that is usually associated with pathological immunological niches: activated neutrophils increase oxygen utilization, while macrophages and lymphocytes enhance glycolysis. Consequently, there is a decrease in the local amount of oxygen and a shift to a hypoxic microenvironment, leading to the activation of HIF signaling and modulation of immune cell activity. This regulatory loop demonstrates the correlation between inflammation, hypoxia, and immune cell metabolism [29].

The final effects of hypoxia on inflamed tissue depend on the cell type and context. In oxygen-deprived inflamed tissue, HIF1-α controls and regulates the activity of myeloid cells as well as T cells, thus influencing innate and adaptive immunity. On one hand, hypoxia promotes the activity of innate immune cells, while on the other hand, it suppresses the adaptive immune system response, to avoid the excessive activation of immune host defense and tissue damage.

In a hypoxic microenvironment, neutrophils, macrophages, and other innate immune cells are generally involved in the primary pathogen’s response and host defense. Hypoxia can act on innate immune cells, activating an anti-inflammatory response. It has been demonstrated that the neutrophils in hypoxia are able to modify the intestinal mucosal microenvironment to resolve inflammation [30]. Hypoxia promotes innate immune cell activity and cell survival. The induction of ATP production, caused by HIF activation, increases the motility, invasiveness, and bactericidal activity of myeloid cells [31,32]. Acknowledging the importance of the oxygen concentration for the outcome of bacterial infection, Thompson et al. demonstrated that the induction of chronic hypoxia before bacterial infection reprograms the innate immune response and modifies leukocytes’ glucose metabolism, leading to improved mouse survival after infection [33]. By contrast, hypoxia can have pro-inflammatory activity. For example, in inflammatory bowel disease, intestinal mucosa is characterized by a severe hypoxia. In this case, inflammation induces a vascular disorder that leads to profound tissue fibrosis [34].

Adaptive or acquired immunity is generally involved in the elimination of molecules, which is known as “non-self”. It is characterized by lymphocytes subdivided into T and B cells. B cells are involved in antibody production, while T cells—including effector T cytotoxic CD8+ cells, helper T CD4+ cells, and regulatory T (Treg) cells—are implicated in the maintenance of self-tolerance. Regarding the role of hypoxia in regulating adaptive immunity, in a context of damaged, inflamed, and strongly hypoxic tissue, the HIF1-α-dependent signaling pathway is able to regulate the immunosuppressive function of Treg cells. Local tissue hypoxia downregulates the immune response by Treg cells and inhibits T effector cells in the tissue microenvironment, avoiding excessive tissue damage [35]. Furthermore, HIF1-α, as the master regulator gene of hypoxia, affects immune cell activities in autoimmune diseases. In this pathological condition, HIF1-α influences the inflammatory response by inducing a switch from T helper cells to Treg cells [36]. Cho et al. examined the correlation between cellular response and limited oxygen levels, hypoxia signaling, and lymphocyte activation and demonstrated that the depletion of HIF-α in CD4+ T cells is able to regulate their production of cytokines, affecting humoral immunity responses and antibody class switching [37].

HIF-α isoforms can be differentially activated in macrophages and exert an antagonistic role in nitric oxide (NO) homeostasis during inflammation. Takeda et al. demonstrated that Th1 cytokines, such as Interferon γ (IFNγ), determine M1 macrophage polarization through the induction of HIF1-α, while Th2 cytokines, such as IL-4 and IL-13, induce HIF2-α expression during M2 activation. HIF1-α takes part in acute phase response through the regulation of inducible nitric oxide synthase (iNOS) expression. On the contrary, HIF2-α acts on the long-term response controlling arginase1 expression in order to suppress NO synthesis [38].

One explanation of the direct link between hypoxia and inflammation is the effects of hydroxylases PHDs and FIHs that are involved with the innate immune system response and adaptive immunity. Scholz et al. demonstrated that hydroxylases play a crucial role in regulating inflammation through affecting the IL1β pathway, and consequently, IL1β-induced NFκB activity. These results suggest the possible therapeutic use of hydroxylase inhibitors as new anti-inflammatory agents [39]. HIF prolyl-4-hydroxylases (HIF-P4H) inhibition exerts a positive influence on the activation and stability of the tumor suppressor p53, which besides positively regulating the expression of pro-apoptotic genes and the activity of the caspases, is known to be a negative regulator of inflammation [40,41]. Ullah et al. showed that the same pathway, negatively regulating the inflammatory response in vitro and in vivo, contributes to the suppression of NFκB activity [42]. The pro-inflammatory role of hypoxia is also due to the direct activation of the NFκB pathway. NFκB is a family of different proteins, encoded by five genes, which generate multiple homo- and hetero-dimeric complexes with different DNA binding affinities. Among them, the most commonly known is the heterodimeric complex p50/p65, which is inactive in the cytosol for the interaction with its regulatory protein IkBα (nuclear factor of kappa light polypeptide gene enhancer in B-cells inhibitor, alpha). NFκB can be activated through a canonical or a non-canonical pathway regulating inflammation, immune responses, proliferation, apoptosis, and angiogenesis [43].

In the canonical pathway, the activation of IKKB kinase induces IkBα phosphorylation and the release of NFκB, which is translocated into the nucleus, where it can activate the transcription of its target genes. It has been demonstrated that under hypoxic conditions, the inhibition of PHDs and FIHs regulates the activity of IKKB, inducing the nuclear translocation of NFκB. Moreover, it has been reported that during microbial infections, hypoxia-mediated NFκB induction controls HIF1-α activity in macrophages, enhancing the production of pro-inflammatory cytokines and chemokines and leading to a more efficient and stronger host defense response [44].

The tumor microenvironment (TME) is a useful model for studying the complex relationship between hypoxia and immune cells, which play a key role in influencing tumor development and will be specifically discussed in this review. However, even though the direct effects of hypoxia on the regulation of the phenotype of tumor cells are not our focus, we briefly refer to some well-known and widely described aspects relating hypoxia to tumors [45,46]. A consistent body of evidence indicates that hypoxic TME can have a dual role in modulating the tumor cell phenotype, since it is associated with a more aggressive phenotype and adverse prognosis, but it can elicit anti-tumorigenic effects [45]. This controversial role is often due to the divergent role that HIF1-α and HIF2-α can play, as has been demonstrated using in vitro models of lung and renal carcinoma [47,48]. Roig et al. showed that HIF1-α deficient cells, but not HIF2-α knockout cells, are characterized by a compensatory upregulation of HIF2-α activity that induces a radioresistant phenotype [47]. Raval et al. demonstrated the opposite roles of HIF1-α and HIF2-α in renal cell carcinoma. HIF1-α activates specifically pro-apoptotic genes, thus inhibiting tumor growth, while HIF2-α promotes tumor growth through the induction of pro-tumorigenic genes such as Cyclin D1 and VEGF [48].

Within the TME, hypoxia can regulate tumor progression not only directly affecting tumor cell properties but also modulating the pro- and anti-tumor functions of tumor-associated immune cells [49,50,51]. Several papers showed that hypoxia and HIF-signaling impair Natural Killer (NK) cell anti-tumor activity. Recently, Ni et al. used single-cell RNA sequencing and the conditional deletion of HIF1-α in NK cells to demonstrate that HIF1-α inhibition restores NK cell anti-tumor activity and inhibits tumor growth [52]. Sarkar et al. discussed the correlation between NK cell-based immunotherapy in multiple myeloma and hypoxia; they demonstrated that hypoxia reduces the expression of the NKG2D receptor and CD16 in NK cells, impairing their cytotoxic activity. This effect can be reverted by IL-2 activation of the NK cells [53]. The PI3K/mTOR pathway, activated in NK cells under hypoxia, induces IL-2 expression and upregulates HIF1-α, affecting NK anti-tumor activity [54]. On the other hand, the deletion of HIF1-α inhibits not only tumor growth but the infiltrative capability of NK cells, thus increasing VEGF bioavailability and promoting tumor progression and metastasis [55]. Furthermore, gene set enrichment analysis showed that the exposure of human NK cells to hypoxia reveals a consensus hypoxia transcriptional profile. This transcriptomic analysis demonstrated that hypoxia can change the expression and release of pro-angiogenetic factors, cytokines, and chemokines, as well as the immune suppressive factors influencing NK cell chemotaxis and the recruitment of specific NK cell subsets at the hypoxic tumor tissue and metastatic sites [56]. The pre-exposure of NK cells to hypoxia, through HIF1-α stabilization, causes cells to adapt toward a cytolytic phenotype and acquire tumor immune-surveillance activity [57].

Even macrophages, which have a critical role in modulating the tumor immune response, are strongly affected by hypoxia and HIF. It has been reported that HIF1-α activation increases the migratory activity of macrophages also, reprogramming their metabolic functions [58]. Hypoxia can induce tumor cells to release chemoattractants, stimulating macrophage infiltration [59]. Hypoxia also induces the M2 polarization of macrophages, promoting the acquisition of a pro-tumoral phenotype [60]. Neutrophil recruitment within the TME and its survival and function are supported by a hypoxic environment and HIFs activation [61,62]. The outcome of this hypoxic effect is still unknown, as cancer-associated neutrophils can induce both tumor suppression and tumor progression.

Hypoxia also regulates the activity of myeloid-derived suppressor cells (MDSCs), which are innate immune cells of bone marrow origin. These cells play a prominent anti-tumor role, since they suppress the activity of other immune cells [63]. While some evidence indicates that hypoxia enhances MDSC function [64,65,66], it has been also reported that HIFs can induce MDSCs to acquire a tumor-suppressing phenotype, supporting the activity of the immune system [67]. Hypoxia and HIF signaling can also affect cells of the adaptive immune system such as T-cells. Under hypoxic conditions, T-cells are induced to differentiate into T helpers (Th17) and Tregs, which are also specifically recruited from circulation by cancer cells and chemokines released by macrophages [68,69,70]. Since Tregs suppress the activity of effector T-cells, hypoxia tends to create an immunosuppressive TME. Moreover, HIF1-α works as a direct inducer of PD-L1 (Programmed Death-Ligand 1) expression in cancer cells, macrophages, dendritic cells, and MDSCs, resulting in further effector T-cell inhibition and enhancing tumor immune escape [71,72]. In contrast to previous findings, it has been reported that HIF1-α stimulates immune activation by positively modulating the function of cytotoxic T-cells and contributing to NK cell activation [73,74].

In summary, hypoxia and HIF signaling are crucial for defining the properties of the immunological niche in both physiological and pathological conditions, and for balancing the multiple activities of immune cells.

## 3. Dual Role of Hypoxia in Acute and Chronic Disease Conditions

We argued that the hypoxic microenvironment and HIF signaling directly and indirectly modulate the immune system’s activities.

Recent investigations have shown that hypoxia and the HIF signaling pathway not only induce disease progression but play a protective role after injury and have the capability of cell recovery in different disease conditions. For this reason, regulation and stabilization of the HIF signaling pathway is proposed as a promising strategy for the treatment of several acute and chronic diseases in organs such as the heart, lung, liver, and kidney.

### 3.1. Heart Diseases

Ischemic heart diseases are a heterogenous group of pathological conditions characterized by low oxygen concentrations and an insufficient perfusion of cardiac tissue. The current treatment of ischemic heart disease consists of myocardium reperfusion to restore blood flow. This procedure, known as ischemia reperfusion injury (IRI), leads to the activation of the inflammatory pathway [75,76].

It is well known that autophagy plays a pivotal role in cellular homeostasis, controlling the clearance of damaged organelles, eliminating misfolded proteins or intracellular pathogens, and recycling cellular components [77]. Autophagy, as well as hypoxia, has a dual role: at the beginning, it plays a protective role to support cell survival, but later, it may participate in cell death [78]. Using a rat model, it was recently demonstrated that mitochondrial autophagy and HIF1-α are involved in IRI. BNIP3, a Bcl2 adenovirus E1B-interacting protein, is a BH3-only protein localized in the mitochondrial outer membrane that plays a pro-apoptotic role [79]. Experiments of BNIP3 overexpression and silencing have demonstrated that during IRI, HIF1-α activates BNIP3, which in turn reduces IRI and promotes myocyte survival through mitochondrial autophagy [80].

Several studies showed that HIF is activated under hypoxic conditions of different heart diseases such as ischemic heart disease or heart failure [81]. Morand et al. demonstrated that chronic exposure to hypoxia induces ischemic ventricular arrhythmias and unexpected cardiac death [82]. Chen et al. found that hypoxia and HIF activation increase BNIP3 expression, thus causing cardiomyocyte death, which is characteristic of ischemia and heart failure [83]. It is not well understood whether HIF stabilization and activation plays a decisive role in myocardial protection from acute ischemia to provide tissue tolerance against ischemic injury.

Ischemic preconditioning (IPC) is a new experimental technique that aims to simulate the preadaptation of myocardium to injury. IPC consists of discontinuous episodes of ischemia and reperfusion to induce the adaptation of the tissue to an eventual ischemia. It has been reported that HIF1-α is crucial during this process. Eckle et al. demonstrated in vivo that HIF1-α activates purinergic signaling through the A2B adenosine receptor pathway, exerting a cardio protective role [84]. Alternatively, HIF can affect the process of IPC by modulating the inflammatory response, e.g., upregulating the circulating levels of anti-inflammatory cytokines, such as interleukin-4 and interleukin-10, and downregulating the levels of the pro-inflammatory cytokines, such as interferon-γ [85].

Ischemic post conditioning (IPostC) is another technique that consists of rapid and brief events of ischemia and reperfusion immediately after an ischemic event. This experimental procedure is clinically relevant because patients come to hospital during or after an ischemic attack. It has demonstrated that the HIF-α signaling pathway is involved in this IPostC. The HIF1-α increase after IPostC directly correlates with a decrease of the infarct size [86,87]. In contrast, HIF2-α can enhance myocardial ischemia tolerance through the positive regulation of its target gene amphiregulin and the activation of the downstream Akt survival signaling pathway [88]. Several experimental models of the downregulation or overexpression of PHDs genes demonstrated the central role of the HIF signaling pathway during IRI. Under hypoxic conditions, the knockdown of PHD3 in cardiomyocytes furtherly stabilizes HIF-α, which counteracts the hypoxia-activated apoptosis through the negative regulation of p53, thus protecting the heart from IRI [89,90]. The knockdown of another PHD, PHD2, by increasing cardiomyocyte viability and cardiac function and significantly reducing infarct size, highlights the protective role of hypoxia in an acute myocardial infarction [91]. The overexpression of PHD3 in hypoxic tissue did not affect cardiac function of resting mice but negatively regulated HIF-α accumulation in the tissue, affecting its response to ischemia [92].

Even if PHD inhibitors (PHI) could be promising therapeutic agents for protecting and/or repairing the heart after ischemia, defining the optimal timing for PHI administration is difficult. In addition to cardiomyocytes, the effects of PHI should be evaluated in other cytotypes involved in cardiac remodeling such as myocytes, immune cells, and vascular cells. Therapeutic HIF stabilization might be dangerous and need to be carefully considered through animal and clinical studies.

In conclusion, recent studies demonstrate the role of hypoxia and HIF signaling in heart disease progression or its cardioprotective role after ischemic heart disease.

### 3.2. Pulmonary Diseases

Pulmonary diseases include acute and chronic conditions in which the HIF-α signaling pathway has a dual role. Acute lung injury (ALI) is a condition characterized by pulmonary edema; this involves the infiltration of protein rich fluid in the alveoli, which dampens the air exchange, resulting in hypoxemia and alveolar hypoxia. Some evidence suggests that alveolar hypoxia can determine an inflammatory response characterized by immune cell infiltration, especially by macrophages, and the subsequent upregulation of inflammatory mediators, such as tumor necrosis factor-α (TNF-α), intercellular adhesion molecule-1 (ICAM-1), macrophage inflammatory protein-1β (MIP-1β), and monocyte chemoattractant protein-1 (MCP-1). These events lead to severe lung injury [93,94].

Chronic hypoxia induces not only ventricular hypertrophy but also pulmonary hypertension (PH). PH is another pulmonary disease caused by a blood pressure increase in the arteries of the lung. It is characterized by systemic inflammation, whereas HIF1-α signaling and its stabilization are crucial for pathogenesis and progression [95]. Some studies have demonstrated that a heterozygous deficiency of HIF1-α or HIF2-α, which regulate chronic hypoxia, protects mice against PH [96,97]. Experiments with the homozygous conditional deletion of HIF1-α, in smooth muscle cells, demonstrated that the deletion of HIF1-α can reduce pulmonary vascular remodeling and PH without affecting ventricular hypertrophy and cardiac remodeling [98]. CD146 is a molecule expressed in pulmonary artery smooth muscle cells, and its expression level indicates the severity of the disease. Recently, Luo et al. demonstrated that the CD146–HIF1-α axis is responsible for vascular remodeling and PH, suggesting that the inhibition of this axis could be an innovative and effective therapeutic strategy against PH [99]. Even HIF2-α is able to affect the pathogenesis of PH. High levels of HIF2-α in lung vascular endothelial cells activate endothelial-to-mesenchymal transition, resulting in vascular remodeling and contributing to pulmonary vascular lesions and PH [100].

However, there are many situations where endogenous pathways, by controlling excessive lung inflammation, contribute to the resolution of different pulmonary diseases. A hypoxic microenvironment and HIF activation can be protective under these conditions. A murine model of alveolar epithelial cells with specific HIF1-α knockdown show less survival time and increased pulmonary edema, demonstrating the main role of HIF1-α in the resolution of lung inflammation in vivo [101]. Several studies confirmed the functional role of HIF1-α in lung injury and explained the mechanism of stabilization and the targets involved. Huang et al. demonstrated that this protective role of HIF-α in ALI is mediated by activating the NOD-like receptor 3 (NLRP3) inflammasome [102]. The HIF protective response in lung and its anti-inflammatory roles could be explained through the purinergic signaling pathway. Indeed, hypoxia and HIF stabilization induce the conversion of ATP/ADP in adenosine. At this point, the adenosine signaling pathway—through nucleoside transporters (ENTs) and by activating the adenosine receptors, Adora2a and Adora2b—leads to a cascade of events that induce lung protection and attenuate ALI [103,104]. Adora2a and Adora2b are also direct target genes of HIF2-α and HIF1-α, respectively, supporting the concept of the central role of hypoxia in lung protection through the adenosine signaling pathway [105,106,107]. Furthermore, the HIF-mediated activation of Adora2b in alveolar epithelial cells is one mechanism responsible for lung protection during ALI [108]. Inflammatory hypoxia, by the activity of inflammatory cells as pulmonary invariant natural killer T (iNKT) cells, determines the hypoxic environment in which there is HIF stabilization [30,109].

Low oxygen levels, supported by pulmonary HIF stabilization, prevent hyperoxic lung injury. Mice with inflammatory lung injury exhibit lower survival rates in higher levels of oxygen than mice in low oxygen level conditions [110]. Even if a high level of oxygen could be useful for patients in the short term, prolonged exposure to high oxygen concentrations causes severe lung injury and increased mortality. This mainly occurs through the inhibition of anti-inflammatory pathways such as Adora2a, contributing to macrophages infiltration [111].

Inflammatory molecules such as endotoxin lipopolysaccharides (LPS) or bacterial compounds could mediate HIF stabilization in ALI. It is well known that during sepsis, LPS is able to activate Toll-like receptors (TLRs), which are responsible for activation of the immune response and inflammatory process in ALI. TLRs, through regulation of the NFκB pathway in both immune and endothelial cells, finally induce the expression of pro-inflammatory genes, such as interleukin-1beta (IL-1β) and interleukin-6 (IL-6) [112] Recently, Wu et al. used a rat model of ALI to clarify the role of sepsis and hypoxia in ALI. The authors demonstrated that hypoxia associated with LPS-mediated inflammation increases the alveolar macrophage cell’s response, affecting the integrity of alveolar capillary membrane. Moreover, LPS, in hypoxic condition, induces HIF1-α accumulation to protect the lung during injury, by upregulating the TLR4 signaling pathway and the inflammatory response [11]. 

In conclusion, experimental evidence has demonstrated that hypoxia and HIF signaling are crucial for the progression of acute and chronic pulmonary disease by playing opposite roles either injuring or protecting tissue.

### 3.3. Liver Diseases

Liver diseases can be classified as acute diseases, such as acute liver failure (ALF), and chronic diseases, such as liver fibrosis or cirrhosis. The hyperactivation of hepatic stellate cells and the overexpression of matrix proteins are the main events that result in the loss of tissue functions up to a cirrhosis condition. 

Hypoxia and HIF stabilization mainly protect against acute diseases, while they have the opposite effect against chronic diseases [113,114]. The protective role of HIF1-α has been demonstrated recently in murine models of acute hepatic inflammation. T-cells-specific deletion of HIF1-α increases neutrophil infiltration and the recruitment of aberrant γδ T-cells into the liver, finally favoring acute hepatic inflammation [115]. Genetic deletion of the HIF2-α gene in myeloid cells reduces the progression of acute liver disease by inducing liver macrophages to overexpress IL-6 and protects against acute injury [116].

The capability of the hypoxia pathway to regulate hepatic damage following ischemia was also investigated in PHD1-deficient mice. The loss of PHD1, plus the downregulation of Keap1 (Kelch-like ECH-associated protein 1), a sensor of oxidative stress, favors the constitutive activation of HIF1-α, protecting hepatocytes against ischemia/reperfusion-induced liver injury and preventing liver fibrosis [117,118]. Finally, the protective role of HIF in the liver can be related to its activation, which is mediated by adenosine receptors, through a mechanism similar to that described for lung injury. Functional studies demonstrated that this pathway can deaden inflammation and facilitate injury resolution and ischemia tolerance [119].

Regarding liver chronic diseases, the role of HIF1-α in fibrosis progression has been described. Mesarwi et al. showed that in hepatocytes, the specific deletion of HIF1-α can protect against liver fibrosis [120]. Moczydlowska et al. demonstrated that the transcriptional activation of HIF1-α is crucial for the establishment and progression of liver fibrosis [121]. Another study showed that the induction of genes involved in epithelial to mesenchymal transition (EMT) is another way through which HIF1-α can promote liver fibrosis progression [122]. Some papers also showed that HIF could play a role in the attenuation of liver fibrosis or in liver regeneration. Schadde et al. used a rat model of liver regeneration to show that hypoxia accelerates liver regeneration [123]. Moreover, Wang et al. demonstrated that VHL overexpression is able to downregulate fibrogenic genes by affecting HIF-α stability, thus reducing liver inflammation and fibrosis [124]. Recently, Dirscherl et al. studied the crosstalk between different cytotypes and found that hypoxia is able to induce VEGF-dependent angiogenesis in hepatic stellate cells, accelerating liver regeneration [125].

### 3.4. Kidney Diseases

Acute kidney injury (AKI) is the most frequent renal disease. It begins with the injury of renal epithelial cells and leads to a rapid loss of tissue function. At first, renal tubular epithelial cells try to dedifferentiate and proliferate to overcome the injury, but when cells can no longer restore the situation and/or the injury become more severe, AKI progression to chronic kidney disease (CKD) is unavoidable [126,127]. The role of hypoxia and HIFs in kidney injury has been widely discussed taking into account the fact that the renal tissue is physiologically characterized by a low oxygen tension and renal tubules have a high consumption of oxygen [128]. In kidney injury and repair, hypoxia responses depend mainly on erythropoiesis, angiogenesis, and anaerobic glucose metabolism. Erythropoietin (EPO) and glycolytic genes are mainly regulated by HIF2-α or HIF1-α, respectively [129,130], while VEGF induction is regulated by HIF1-α and HIF2-α [131].

Many studies have focused on the inhibitory role of the hypoxia signaling pathway in kidney injury progression, highlighting its positive role in kidney protection or repair. Shu et al. discussed this topic considering results from in vivo experiments on mouse and rat models [128]. Here, we present a brief summary of the latest studies.

By using an in vitro and in vivo renal ischemia model, it has been demonstrated that HIF1-α stabilization, induced by PHD inhibition, protects the kidney from ischemia by upregulating glycogen synthesis [132]. Moreover, the protective role of PHD inhibitors has been reported in both CKD and AKI. By inducing the stabilization of HIF1-α, these inhibitors can restore capillary density, thus reducing cardiovascular complications; reduce pro-inflammatory responses and apoptosis; and induce macrophage infiltration, conferring protection against ischemic renal injury [133,134,135].

Xie et al. recently demonstrated that the inhibition of PDH activity ameliorated glomerular endothelial cells (GECs) injury associated with diabetic nephropathy. This effect was related to the glucose metabolism increase due to the HIF1-αmediated transcription of genes associated with glycolysis [136]. Anemia is one of the consequences of advanced CKD. Additional studies enabled the introduction of PHD inhibitors in clinical trials for the treatment of anemia associated with chronic diseases. PHD inhibitors are able to stimulate renal and hepatic erythropoiesis. Among them, Roxadustat (FG-4592) has completed phase II clinical trials successfully and is now in phase III trials [137]. Phase III clinical trials are ongoing with another carboxylic acid-based PHD inhibitor, Vadadustat (AKB-6548) [138]. These are the first examples of clinical trials with compounds that target HIF signaling. These data clearly demonstrate the central role of hypoxia in physiology, above all during the progression of different pathologies.

Recently, different epigenetic mechanisms as well as miRNA activities have been described as players in I/R injury. Wei et al. suggested another possibility through which HIF1-α guarantees a protective role following kidney injury. The authors demonstrated that after ischemia in renal tubules, HIF1-α upregulates different miRNAs. Among them, miR688 is upregulated in patients with AKI and inhibits cell mitochondrial fragmentation, facilitating kidney protection [139]. Other studies directly correlated the activity of miR21 with the protective role of HIF1-α in renal I/R injury. Xu et al. demonstrated that in hypoxic conditions, HIF1-α activation determines miR21 upregulation, inducing angiogenesis and leading to renal protection [140]. Finally, Song et al. demonstrated, both in vitro and in vivo, that miR21 increases HIF1-α and HIF2-α expression through the PTEN/AKT/mTOR pathway, thus protecting epithelial cells against injury [141].

## 4. Conclusions

Hypoxia is associated with several physiological or pathological conditions and plays a primary role in determining the fate of cells and tissue.

The cellular response to hypoxia consists of the activation of multiple genes involved in different biological processes; the master regulatory factor mediating the cellular response to this condition is the hypoxia-inducible factor (HIF). Here, we reported recent studies that showed the main mechanisms through which hypoxia and HIF exert a double role. We discussed how hypoxia signaling and HIF activation can have anti- and pro-inflammatory effects regulating the activity of immune system cells. Moreover, other studies showed that hypoxia and the HIF signaling pathway play a double role in acute and chronic diseases. Hypoxia and the HIF signaling pathway not only induce disease progression but can play a protective role after injury and promote cell recovery in different disease conditions.
